# Clinicopathological and Prognostic Characteristics of CD276 (B7-H3) Expression in Adrenocortical Carcinoma

**DOI:** 10.1155/2020/5354825

**Published:** 2020-01-08

**Authors:** Jiayu Liang, Zhihong Liu, Tianjiao Pei, Yingming Xiao, Liang Zhou, Yongquan Tang, Chuan Zhou, Kan Wu, Fuxun Zhang, Fan Zhang, Xiaoxue Yin, Ni Chen, Xin Wei, Yiping Lu, Yuchun Zhu

**Affiliations:** ^1^Institute of Urology, Department of Urology, West China Hospital, Sichuan University, Chengdu, Sichuan, China; ^2^Department of Obstetrics and Gynecology, West China Second University Hospital of Sichuan University, Chengdu, Sichuan, China; ^3^Department of Urology, Sichuan Cancer Hospital, Chengdu, Sichuan, China; ^4^Department of Pediatric Surgery, West China Hospital, Sichuan University, Chengdu, Sichuan, China; ^5^Department of Pathology, West China Hospital, Sichuan University, Chengdu, Sichuan, China

## Abstract

**Background:**

Adrenocortical carcinoma (ACC) is a rare malignant endocrine tumor with a high tumor recurrence rate and poor postoperative survival. Recent studies suggest that CD276- (B7-H3) targeted therapy represents a promising therapeutic option for solid tumors. However, little is known about the expression status of CD276 or its association with progression and prognosis of ACC.

**Methods:**

Clinical data were retrospectively analyzed from patients who underwent resection of ACC at our institution (*n* = 48). Archived, formalin-fixed, and paraffin-embedded samples were collected for immunohistochemical analysis, and the correlation between CD276 expression and clinicopathological parameters was evaluated. Kaplan–Meier and univariate/multivariate Cox regression methods were implemented to identify any prognostic effects. Data from The Cancer Genome Atlas (TCGA) ACC cohort (*n* = 77) were retrieved for quantitative validation analysis.

**Results:**

Positive expression of CD276 was detected on the cell membrane and in the cytoplasm of cancer cells or tumor-associated vascular cells in 91.67% (44/48) of ACCs. Vascular expression of CD276 was associated with local aggression (higher T stage, *P* = 0.029) and advanced ENSAT stage (*P* = 0.02). Specifically, patients with a higher CD276-positive cancer cell density exhibited significantly worse overall survival and recurrence-free survival in our cohort (HR = 2.8, *P* = 0.01, and HR = 7.52, *P* < 0.001, respectively) and in the validation cohort (HR = 2.4, *P* = 0.033, and HR = 3.7, *P* < 0.001, respectively). The prognostic association remained significant in multivariate Cox regression analysis. Further analysis indicated that CD276 participates in regulating the immune response as well as in the malignant biological behaviors of ACC.

**Conclusion:**

These findings highlight the immune checkpoint factor CD276 as an independent prognostic factor and a potential therapeutic target in ACC.

## 1. Introduction

Adrenocortical carcinoma (ACC) is a rare endocrine malignancy (0.5-2 cases per million per year) with a heterogeneous and often poor prognosis [1, 2]. Patients are often diagnosed at an advanced stage. While surgical resection remains the first option, nearly 50% of ACC patients who undergo initial complete resection develop recurrent or metastatic disease [3]. Tumor stage is determined according to the European Network for the Study of Adrenal Tumors' (ENSAT) classification of TNM stages [4], resection (R) status [5, 6], Ki67 index [7], and a set of newfound biomarkers [8] that represent the known prognostic factors.

Both oncogenesis and immune status are poorly understood in ACC. In the tumor microenvironment, the immunosuppressive and immunostimulating signatures have a potential prognostic value for some cancer types [9, 10]. Recently, Liu et al. reported that CD8^+^ T cells and expression of programmed death ligand 1 (PD-L1/B7-H1) were significantly associated with improved survival, indicating a potential role for the immune signature in the assessment of ACC prognosis [11]. However, PD-L1 is reportedly only expressed in approximately 10% of ACC tumor cells and cell membranes [12, 13]. Given that the current immunotherapy (PD-L1 inhibitor avelumab) failed in a phase I clinical trial for ACC [14], identification of novel immune markers and therapeutic targets in ACC is urgently needed.

CD276 (B7-H3) is one of the B7 superfamily molecules that correlates with prognosis in various cancer types [15, 16]. As an emerging immune checkpoint, factor, CD276 has recently been identified as a promising candidate target in multiple cancers. Increasing data suggest that inhibition of CD276 may suppress tumor growth [17], and CD276-targeted therapy has shown broad tumoricidal and antimetastatic activity in vivo [18]. Additionally, a preclinical study on B7-H3-targeted CAR T cells revealed antitumor activities in solid tumors [19]. Despite these advancements, our knowledge of the expression patterns of CD276 in ACC is lacking. Whether CD276 is associated with the prognosis of ACC remains unclear.

In the current study, we aimed to evaluate the clinical significance of CD276 as an emerging immune checkpoint in ACC. The relationship between CD276 and multiple clinicopathological parameters was explored. We demonstrated that differential expression patterns of CD276 were closely associated with tumor progression and prognosis in ACC patients. Herein, the regulatory relationships between CD276 and the immune signature are revealed to improve the understanding of the role of CD276 in the ACC microenvironment.

## 2. Patients and Methods

### 2.1. Patient Cohort

Between 2009 and 2016, patients who underwent tumor resection at the West China Hospital that were pathologically confirmed as ACC were analyzed. A total of 48 patients were included in this study. Related clinical records were extracted as per our previous report [20], including gender, age, grade, stage, treatment, R status, Ki67 index, and clinical follow-up data. Corresponding formalin-fixed, paraffin-embedded (FFPE) tissues were retrospectively collected from our institutional biobank. Under the ethical guidelines as required by the Declaration of Helsinki, informed consent was provided by each patient, and the research protocol was approved by the West China Hospital of Sichuan University Biomedical Research Ethics Committee.

### 2.2. Immunohistochemistry and Image Analysis

Serial FFPE tissue sections with a thickness of 4 *μ*m were subjected to immunohistochemistry (IHC) analysis following protocols. Briefly, sections were deparaffinized in xylene and rehydrated through a graded ethanol series, followed by placement in 3% H_2_O_2_ for 15 min at room temperature. After heat-mediated retrieval using sodium citrate or EDTA, slides were incubated with primary antibody overnight at 4°C. The primary antibody used was a rabbit antihuman B7-H3 (D9M2L) XP® monoclonal antibody (#14058, Cell Signaling Technology, Danvers, MA, USA). SignalStain® Boost IHC Detection Reagent (HRP, Rabbit, CST) was applied for 30 min at room temperature according to the manufacturer's instructions.

The immunostaining results were independently evaluated by two investigators blinded to the clinical data (X.Y. and N.C.). The semiquantitative H-score of the cytoplasmic staining intensity was calculated as 0 (negative), 1 (weak), 2 (moderate), or 3 (strong). Due to the limited number of ACC cases in this cohort, we next merged them into high expression (strong and moderate expression) and low expression (negative and weak expression) groups. Membranous and vascular expression status was evaluated as “positive” or “negative”. The cut-off proportion of positive expression was 5% in each specimen.

### 2.3. Validation Data and Analysis Tools

ACC clinical data and RNA-Seq data from the TCGA project were retrieved from the UCSC Xena project (http://xena.ucsc.edu). This study meets the publication guidelines provided by TCGA (https://cancergenome.nih.gov/publications/publicationguidelines). One-way ANOVA and the log-rank test were used in the GEPIA analysis [21].

### 2.4. Gene Set Enrichment Analysis (GSEA)

Gene expression relationships were evaluated using the R system, and the coexpression cut-off was Pearson ∣*R*∣ > 0.4. Next, GSEA was performed using GSEA v3.0 (http://www.broadinstitute.org/gsea/). The gene sets used in this work were downloaded from the Molecular Signatures Database (https://software.broadinstitute.org/gsea/msigdb/index.jsp). GO terms with a *P* value <0.05 and an enrichment score > 1.0 were considered significant [22].

### 2.5. Statistical Analysis

Statistical analyses were performed using the R system (version 3.4.4) and GraphPad Prism version 6.02 (GraphPad Software, La Jolla). Overall survival (OS) was defined as the time elapsed from primary resection of ACC to death due to any cause. Disease-free survival (DFS, also called relapse-free survival) was defined as the time elapsed from primary resection of ACC to the first recurrence (locoregional or systemic). As per our previous report, recurrent disease was diagnosed based on clinical, radiographic, and laboratory evidence, including local recurrence, peritoneal carcinomatosis, and distant metastases. The Chi-square test or Fisher's exact test was used to compare clinicopathological variables between two groups. Survival analyses were analyzed by the Kaplan–Meier method and log-rank test. Univariate and multivariate Cox regression analyses were performed to identify significant risk factors, and variables with a *P* value <0.05 were included in the multivariate Cox regression. *P* values <0.05 were considered statistically significant.

## 3. Results

### 3.1. Differential Expression of CD276 in ACC Tissues

**Figure 1:**
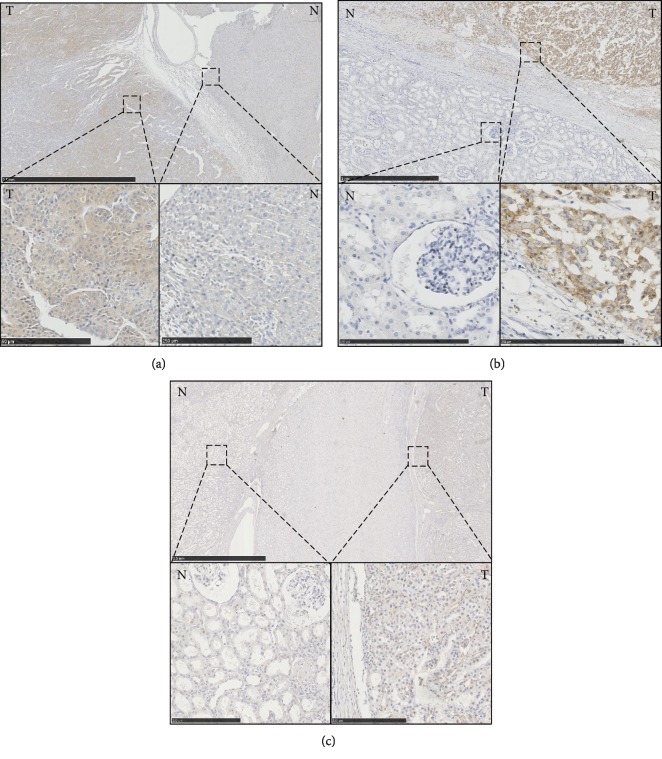
The expression of CD276 in different tissues: (a) left: ACC tissues, right: adjacent normal adrenal tissues; (b) right: ACC tissues, left: adjacent normal renal tissues; (c) right: Renal cell carcinoma tissues, left: adjacent normal tissues. T: tumor; N: normal tissues. Scale bar (bottom): 250 *μ*m.

**Figure 2:**
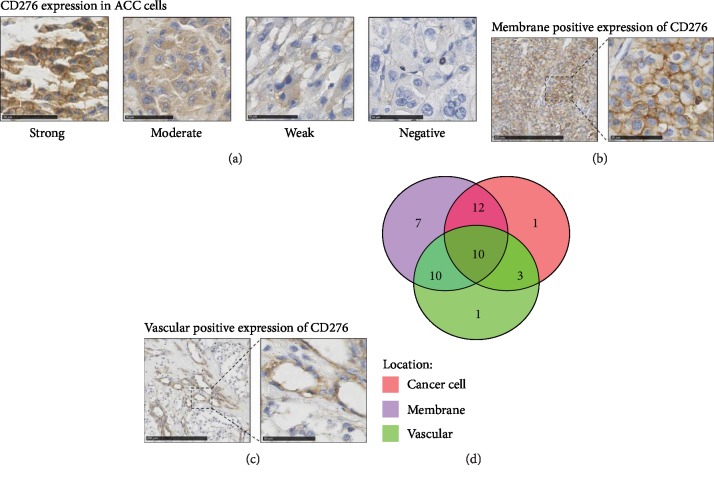
Differential CD276 expression in adrenocortical carcinoma. (a) Immunohistochemistry (IHC) results of CD276 expression in ACC cells. Representative staining intensities of scores 3 (strong), 2 (moderate), and 1 (weak) and 0 (negative). Scale bar: 50 *μ*m. (b) Representative staining of CD276-positive cell membrane localization. Scale bar: left = 250 *μ*m, right = 50 *μ*m. (c) Representative staining of the CD276-positive tumor vasculature. Scale bar: left = 250 *μ*m, right = 50 *μ*m. (d) The number of positive cases with different expression patterns (*n* = 44).

ACC cases from the West China Hospital cohort (*n* = 48) from 2009 to 2016 were collected, and their clinical and pathological characteristics were analyzed (Table 1). Immunohistochemical (IHC) detection of CD276 was performed in these cases. Renal cell carcinoma tissue was used as positive control and adjacent normal renal tissue was negative control. We found that expression of CD276 in adjacent normal adrenal tissues (Figure 1(a)) and adjacent normal renal tissues (Figures 1(b) and 1(c)) was negative in both types of tissues. For ACC tissues, the overall positive rate of CD276 was 91.67% (44/48). Differential expression of CD276 in tumor cells was scored as 0 (negative), 1 (weak), 2 (moderate), and 3 (strong). Representative sections with different staining intensity grades of CD276 are shown in Figure 2(a). Among them, moderate and strong staining of CD276 accounted for 54.17% (26/48) of cases. CD276 expression was also detected in the membrane of tumor cells (Figure 2(b)). Furthermore, 81.25% of ACC samples exhibited positive membranous expression of CD276, while only 9 cases were identified as membranous CD276-negative ACC. A subset of cases (50%) exhibited positive localization of CD276 in the ACC-associated vasculature (Figure 2(c)). The expression patterns of CD276 are summarized in Figure 2(d).

### 3.2. Association between CD276 Expression and the Clinicopathological Characteristics of ACC

Based on the classification of the European Network for the Study of Adrenal Tumors (ENSAT), local tumor infiltration and invasion status were evaluated as well as other clinicopathological parameters. Next, the potential correlation between the differential expression patterns and the intensity of CD276 and pathological features was compared (Table 1). ACC patients were divided into subgroups according to their differential CD276 expression patterns (strong/moderate vs. weak/negative intensity in tumor cells; positive vs. negative tumor cell membranous location; positive vs. negative expression in tumor vasculature).

**Figure 3:**
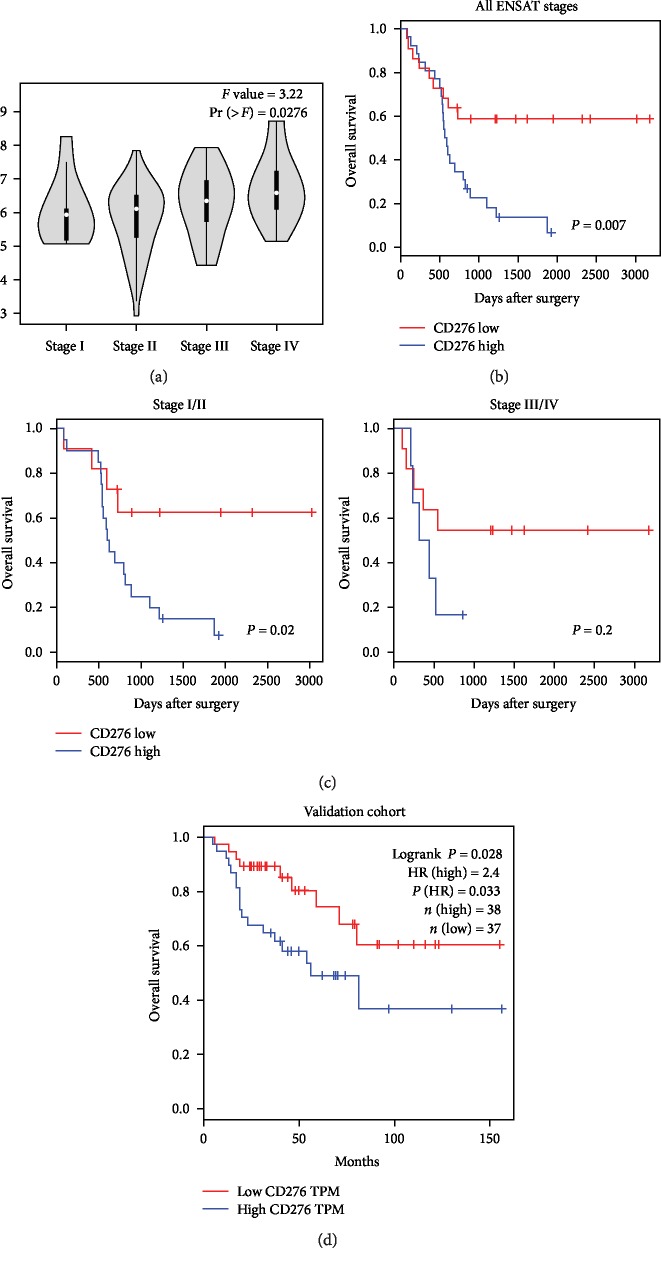
Association between CD276 and overall survival of ACC. (a) Differential distributions of CD276 mRNA expression in stages I-IV in the validation cohort (*P* = 0.0276). (b) The correlation between cytoplasmic CD276 expression score and overall survival of ACC (all ENSAT stages, *P* = 0.007). (c) The correlation between the cytoplasmic CD276 expression score and overall survival of ACC (left: ENSAT stages I and II, *P* = 0.007; right: ENSAT stages III and IV, *P* = 0.2). (d) The correlation between the CD276 mRNA expression level and overall survival of ACC in the validation cohort (*P* = 0.028).

Both tumor cell expression and vascular expression of CD276 were differentially distributed in different gender groups (*P* = 0.01 and *P* = 0.003, separately). Membrane expression of CD276 was lower in cases with larger tumors (≥7.5 cm, *P* = 0.022). Interestingly, expression of CD276 in the tumor vasculature was significantly correlated with gender, age, T stage, and ENSAT stage in our patient cohort, suggesting that the CD276-invasive rate in the tumor vasculature was higher in either male ACC patients (*P* = 0.003) or older ACC patients (≥65, *P* = 0.044). Positive expression of CD276 in the tumor vasculature may also indicate a higher risk of local tumor infiltration, adjacent organ invasion or venous tumor thrombus (*P* = 0.029), and advanced ENSAT stage (*P* = 0.020). However, there was no association observed between hormone secretion, N stage, or Ki67 index and the CD276 expression patterns. In addition, mRNA expression of CD276 was also found to correlate with the disease stage in the validation cohort (*P* = 0.0276, Figure 3(a)).

### 3.3. CD276 Expression and Overall Survival in ACC

Next, we explored the association between CD276 expression and overall survival of ACC patients. In the Kaplan–Meier analysis, ACC cases with a higher intensity of CD276 expression in tumor cells exhibited significantly poorer overall survival compared to those with lower CD276 expression levels (*P* = 0.007, Figure 3(b)). However, neither membranous nor vascular expression of CD276 was correlated with OS. We further performed univariate and multivariate Cox analyses to examine the prognostic effect of CD276 expression (Table 2). Gender, age, hormone secretion, laterality, tumor size, T stage, N stage, ENSAT stage, R status, Ki67 index, and all 3 different expression patterns of CD276 were included in the regression model. As a result, the higher Ki-67 index (HR = 3.16, 95% CI: 1.52-6.61, *P* = 0.002) and higher intensity of CD276 expression in tumor cells (HR = 2.8, 95% CI: 1.28-6.15, *P* = 0.01) were the only prognostic factors in the multivariate Cox model, suggesting that differential expression of CD276 in tumor cells is an independent OS factor in ACC, as well as the Ki-67 index.

Meanwhile, it is important to note that the prognostic effect of CD276 was also observed in earlier ENSAT stages (*P* = 0.02, Figure 3(c)) in our cohort. To further assess its prognostic correlation, we examined the mRNA expression data of CD276 and the OS time using data retrieved from the TCGA-ACC cohort. Similar to our cohort, the prognostic effect of CD276 mRNA expression was verified in the validation dataset (HR = 2.4, *P* = 0.033, Figure 3(d)).

### 3.4. CD276 Expression and Disease Recurrence of ACC

**Figure 4:**
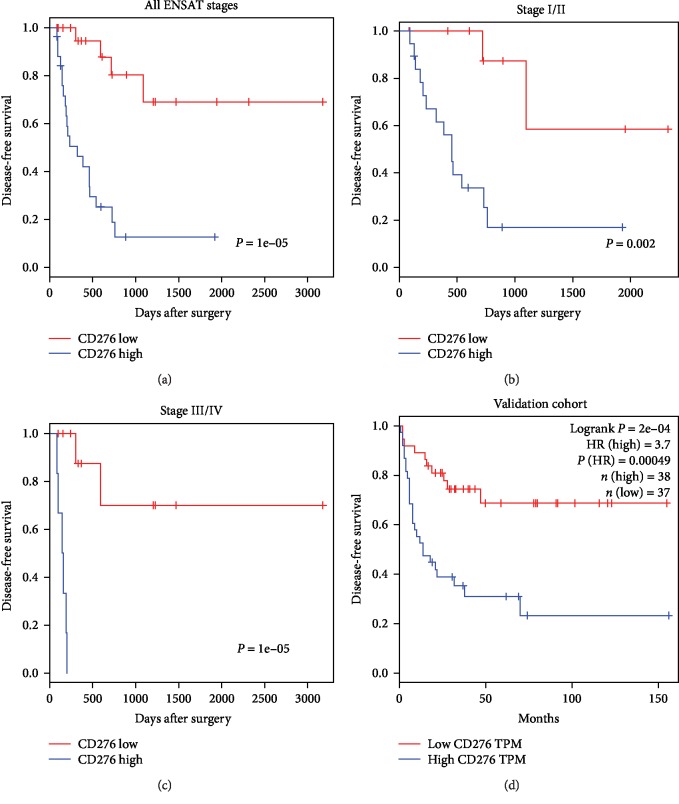
Association between CD276 and disease-free survival of ACC. (a) The correlation between the cytoplasmic CD276 expression score and disease-free survival of ACC (all ENSAT stages, *P* < 0.001). (b) The correlation between the cytoplasmic CD276 expression score and disease-free survival of ACC (ENSAT stages I and II, *P* = 0.002). (c) The correlation between the cytoplasmic CD276 expression score and disease-free survival of ACC (ENSAT stages III and IV, *P* < 0.001). (d) The correlation between the CD276 mRNA expression level and disease-free survival of ACC in the validation cohort (*P* < 0.001).

Given the high recurrence rate of ACC, we next assessed whether CD276 expression is associated with tumor recurrence after surgical resection. The results demonstrated that higher expression of CD276 in tumor cells, but not membrane localization or vascular expression, was significantly correlated with RFS (*P* < 0.001, Figure 4(a)). More importantly, the recurrence-related effects of CD276 were also apparent in both ACC subgroups of earlier (I/II, *P* = 0.002, Figure 4(b)) and advanced ENSAT (III/IV, *P* < 0.001, Figure 4(c)) stages. Multivariate Cox regression modeling suggested that surgical assessment (R1/2/X, HR = 2.8, 95% CI: 1.23-6.39, *P* = 0.014) and CD276 expression in tumor cells (HR = 7.52, 95% CI: 2.47-22.91, *P* < 0.001) were independent recurrence risk factors for ACC (Table 2). In the validation cohort, CD276 was also found to significantly correlate with RFS (HR = 3.7, *P* value = 0.00049, Figure 4(d)). These findings indicate that high expression of the immune checkpoint factor CD276 in tumor cells is a recurrence risk factor for ACC patients.

### 3.5. CD276-Related Signatures in the Immune Response and Tumor Development of ACC

To explore the biological role of CD276 in ACC, we analyzed the molecular signature of CD276 using gene set enrichment analysis (GSEA). First, genes that strongly coexpressed with CD276 were selected (ranked by Pearson |*R*|) from the TCGA-ACC dataset. As a result, we found that genes that highly correlated with CD276 expression were more involved in immune signatures, including “immune system,” “adaptive immune system,” “innate immune system,” and “cytokine signaling in immune system” (Supplemental [Supplementary-material supplementary-material-1]). Through analyzing immune-related genes from the most significantly enriched gene set, “immune system,” an obvious enrichment landscape of these signatures in higher CD276 expression cases was observed (Supplemental [Supplementary-material supplementary-material-1]). Subsequent functional analysis of the CD276-correlated genes suggested that, except for immune response-related functions, such as the T cell receptor signaling pathway, antigen processing and presentation pathway, and stimulatory C-type lectin receptor signaling pathway, CD276 also participated in cell proliferation and the negative regulation of apoptosis of ACC cells (Supplemental [Supplementary-material supplementary-material-1]). These results indicate that CD276 is closely related to both immune regulation and tumor development in ACC.

## 4. Discussion

Based on two large, independent ACC cohorts, we performed the first study exploring the link between the differential expression patterns of CD276 and the clinical characteristics of adrenocortical carcinoma patients. Herein, expression of the CD276 protein was observed in more than 90% of cases with this extremely rare and high malignant carcinoma. Our findings reveal the prognostic significance of CD276 in ACC.

As a member of the B7/CD28 superfamily and immune checkpoint family, CD276 (B7-H3) plays an important role in the microenvironment between tumors and the host immune system. Negative regulation by CD276 of the immune cell response, such as T cells and NK cells, has recently been reported [23, 24]. Meanwhile, CD276 is also related to invasiveness and the epithelial-to-mesenchymal transition pathway in cancer cells [16]. CD276 expression has been reported in a number of malignancies in the genitourinary system, gastrointestinal system, and respiratory system [25–32]. The prognostic effects of CD276 have also been demonstrated in clear cell renal cell carcinoma [25], prostate cancer [28], colorectal cancer [26], and NSCLC [29]. Similar to these studies, our findings demonstrated a close association between high CD276 expression and an increased risk of recurrence and poor overall survival in ACC patients who underwent surgical resection. Moreover, we further observed a significant increase in vascular expression of CD276 in ACC cases with more aggressive tumor features (advanced T stage and ENSAT stage), which is consistent with a previous report on distinct cancer types [33].

In recent years, immunotherapy has been widely used to treat various cancer types. Accordingly, the expression of immune checkpoint factors is widely accepted as a predictor of response to immune checkpoint inhibitors. Given the limited expression of PD-L1 in ACC, exploring new targets for ACC patients is urgent. Increasing data suggest that CD276 represents a novel therapeutic immune checkpoint. Inhibition of CD276 is able to suppress tumor growth [17], and CD276-targeted therapy has also shown broad tumoricidal and antimetastatic activity in vivo [18]. In this study, we detected high positive expression of CD276 in ACC tissues, including in tumor cells and the tumor vasculature. These results indicate that CD276 may represent a potential therapeutic target in ACC. In addition, the newly revealed significant association between CD276 expression and clinicopathological features may be helpful in distinguishing patients with a higher CD276 expression status.

The current study is a relatively large single-center cohort study of patients with rare ACC. However, due to the limited number of ACC samples available, full-quantitative experiments in our ACC cohort were not performed. To overcome this limitation, we retrieved external quantitative data from the TCGA cohort in this study, successfully validating the prognostic effects of CD276. The clinical pathological characteristics of the validation cohort were summarized in a previous report [34]. Clinically, multiple parameters have reportedly been correlated with patient prognosis, such as age, hormone secretion, Weiss score, Ki67 index, and resection (R) status [35–40]. Libé et al. analyzed advanced ACC in an ENSAT dataset, demonstrating that GRAS (Grade, R status, age, and symptoms) parameters successfully stratified differential patient prognosis [41]. In our analyses, CD276 correlated with poor survival of ACC patients, and this association remained significant in the multivariate model. The results further indicate the importance of understanding the CD276-regulated immune response and tumor aggressive behaviors in future studies.

In conclusion, in this study, we demonstrate for the first time the clinical significance of CD276 expression in ACC cells and the tumor vasculature. These findings highlight CD276 as an independent prognostic factor and potential immune checkpoint therapeutic target in ACC treatment.

## Figures and Tables

**Table 1:** CD276 expression correlates with clinicopathological characteristics of adrenocortical carcinoma.

Characteristics	All	Cancer cells			Membrane expression			Vascular expression		
		High	Low	*P*	Positive	Negative	*P*	Positive	Negative	*P*
Gender										
Female	31	21	10	0.01^∗^	23	8	0.13	10	21	0.003^∗^
Male	17	5	12		16	1		13	4	
Age										
<65	41	23	18	0.68	32	9	0.32	17	24	0.044^∗^
≥65	7	3	4		7	0		6	1	
Hormone secretion										
No	28	15	13	0.92	23	5	1	12	16	0.41
Yes	20	11	9		16	4		11	9	
Tumor size (cm)										
<7.5	24	14	10	0.56	23	1	0.022^∗^	9	15	0.14
≥7.5	24	12	12		16	8		14	10	
T stage										
T1+T2	33	20	13	0.18	28	5	0.43	13	20	0.029^∗^
T3+T4	15	6	9		11	4		11	4	
Node stage										
N0	40	23	17	0.44	32	8	1	17	23	0.13
N1	8	3	5		7	1		6	2	
ENSAT stage										
I+II	31	20	11	0.052	26	5	0.70	11	20	0.020^∗^
III+IV	17	6	11		13	4		12	5	
Ki67 index										
<20%	29	15	14	0.67	24	5	1	14	15	0.95
≥20%	19	11	8		15	4		9	10	

^∗^Statistical significance (*P* < 0.05).

**Table 2:** Univariate and multivariate Cox analysis of CD276 expression.

	Univariate analysis			Multivariate analysis		
Variables	Hazard ratio	95% CI	*P*	Hazard ratio	95% CI	*P*
OS						
Gender (male)	0.82	0.39-1.73	0.601			
Age (≥65)	1.37	0.52-3.57	0.522			
Hormone secretion (yes)	0.76	0.37-1.55	0.445			
Laterality (right)	0.91	0.45-1.86	0.796			
Tumor size (≥7.5)	0.76	0.38-1.52	0.431			
T stage (T3+T4)	1.14	0.52-2.48	0.741			
N stage (N1)	0.76	0.27-2.16	0.605			
Stage (III+IV)	1.06	0.5-2.25	0.88			
R status (R1/2/X)	1.22	0.6-2.47	0.589			
Ki67 index (high)	3.17	1.53-6.56	0.002	3.16	1.52-6.61	0.002
CD276 tumor cells (high)	2.83	1.29-6.17	0.009	2.8	1.28-6.15	0.01
CD276 membrane (high)	1.4	0.54-3.66	0.488			
CD276 vascular (positive)	1.26	0.63-2.53	0.517			
DFS						
Gender (male)	0.64	0.26-1.54	0.317			
Age (≥65)	0.55	0.13-2.36	0.424			
Hormone secretion (yes)	1.07	0.47-2.41	0.873			
Laterality (right)	1.6	0.71-3.6	0.253			
Tumor size (≥7.5)	0.8	0.36-1.81	0.596			
T stage (T3+T4)	1.62	0.69-3.81	0.272			
N stage (N1)	0.43	0.1-1.83	0.252			
Stage (III+IV)	1.2	0.51-2.83	0.678			
R status (R1/2/X)	3.27	1.44-7.39	0.005	2.8	1.23-6.39	0.014
Ki67 index (high)	0.85	0.35-2.06	0.72			
CD276 tumor cells (high)	8.13	2.71-24.4	<0.001	7.52	2.47-22.91	<0.001
CD276 membrane (high)	0.84	0.31-2.28	0.736			
CD276 vascular (positive)	1.15	0.52-2.56	0.733			

## Data Availability

The data that support the findings of this study are available on request from the corresponding author. The data are not publicly available due to privacy or ethical restrictions.

## Supplementary Materials

Supplementary Materials Supplemental Figure 1: CD276-related signatures in ACC. (A) Gene set enrichment analysis (GSEA) of genes that strongly coexpressed with CD276. The representative gene sets are “immune system,” “adaptive immune system,” “innate immune system,” and “cytokine signaling in immune system”. (B) Heat map of genes from the GSEA “immune system” gene set. The median expression value was used as a cut-off of high expression (red) and low expression (green) of genes. (C) CD276-related functional pathways enriched in ACC. The *Q* value is shown as dots with different colors. The number of enrichment genes is shown as dots with different sizes. The ratio of the coexpressed gene number and the number of annotated genes in each pathway were calculated as the rich factor.
