# Supplementary material for: Clinicopathological and Prognostic Characteristics of CD276 (B7-H3) Expression in Adrenocortical Carcinoma
Source: Dis Markers. 2020 Jan 8;2020:5354825. doi: 10.1155/2020/5354825 (PMC6977319; doi:10.1155/2020/5354825)
Supplement: Supplementary Materials — Supplemental Figure 1: CD276-related signatures in ACC. (A) Gene set enrichment analysis (GSEA) of genes that strongly coexpressed with CD276. The representative gene sets are “immune system,” “adaptive immune system,” “innate immune system,” and “cytokine signaling in immune system”. (B) Heat map of genes from the GSEA “immune system” gene set. The median expression value was used as a cut-off of high expression (red) and low expression (green) of genes. (C) CD276-related functional pathways enriched in ACC. The Q value is shown as dots with different colors. The number of enrichment genes is shown as dots with different sizes. The ratio of the coexpressed gene number and the number of annotated genes in each pathway were calculated as the rich factor. [file 5354825.f1.pdf]

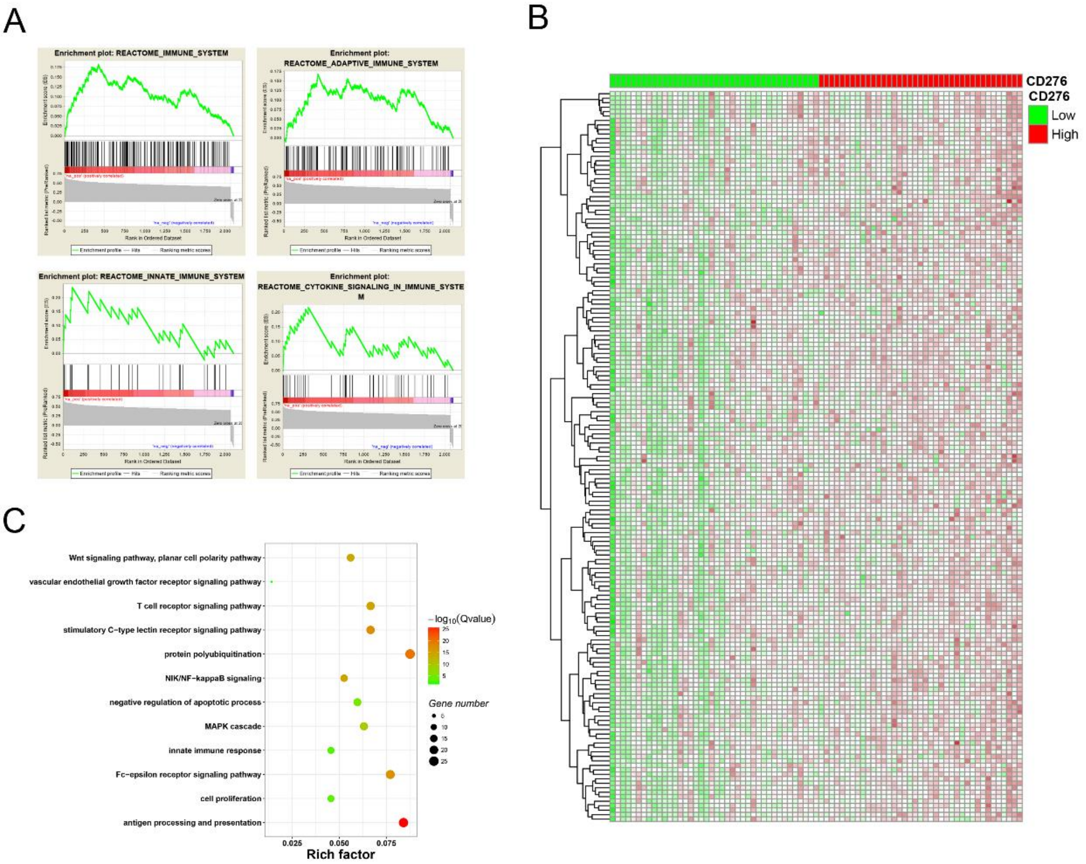

Supplemental Figure 1. CD276-related signatures in ACC. (A) Gene set enrichment analysis (GSEA) of genes that strongly coexpressed with CD276. The representative gene sets are “immune system”, “adaptive immune system”, “innate immune system” and “cytokine signaling in immune system”. (B) Heat map of genes from the GSEA “immune system” gene set. The median expression value was used as a cut-off of high expression (red) and low expression (green) of genes. (C) CD276-related functional pathways enriched in ACC. The Q value is shown as dots with different colors. The number of enrichment genes is shown as dots with different sizes. The ratio of the coexpressed gene number and the number of annotated genes in each pathway was calculated as the rich factor.
